# An Improved Online Fast Self-Calibration Method for Dual-Axis RINS Based on Backtracking Scheme

**DOI:** 10.3390/s22135036

**Published:** 2022-07-04

**Authors:** Jing Li, Lichen Su, Fang Wang, Kailong Li, Lili Zhang

**Affiliations:** 1 Information Engineering College, Beijing Institute of Petrochemical Technology, Beijing 102617, China; bipt_lijing@bipt.edu.cn (J.L.); zhanglili@bipt.edu.cn (L.Z.); 2 School of Automation Science and Electrical Engineering, Beihang University, Beijing 100083, China; 20373546@buaa.edu.cn; 3 China Intelligent Transpation Systems Association, Beijing 100070, China; li.kl@its-china.org.cn; 4 Xufeng Technology Co., Ltd., Yinchuan 750011, China

**Keywords:** inertial measurement unit (IMU) calibration, strapdown inertial navigation system (SINS), Kalman filter, gradient descent

## Abstract

In the field of high accuracy dual-axis rotational inertial navigation system (RINS), the calibration accuracy of the gyroscopes and accelerometers is of great importance. Although rotation modulation can suppress the navigation error caused by scale factor error and bias error in a static condition, it cannot suppress the scale factor errors thoroughly during the maneuvering process of the vehicle due to the two degrees of rotation freedom. The self-calibration method has been studied by many researchers. However, traditional calibration methods need several hours to converge, which is unable to meet the demand for quick response to positioning and orientation. To solve the above problems, we do the following work in this study: (1) we propose a 39-dimensional online calibration Kalman filtering (KF) model to estimate all calibration parameters; (2) Error relationship between calibration parameters error and navigation error are derived; (3) A backtracking filtering scheme is proposed to shorten the calibration process. Experimental results indicate that the proposed method can shorten the calibration process and improve the calibration accuracy simultaneously.

## 1. Introduction

Rotational inertial navigation systems (RINS) have reached high accuracy of navigation in recent years [1,2]. A RINS consists of three gyroscopes, three accelerometers, and one rotational table. the inertial measurement unit (IMU) is installed inside the two-axis rotation table [3]. The accuracy of RINS highly depends on the accuracy of the accelerometers and gyroscopes, hence, the calibration parameters of the IMU are very important [4]. The RINS makes the online self-calibration of IMU a real, self-calibration is an effective way to improve the navigation performance of RINS [5].

Many researchers have studied the INS calibration method [6,7,8,9]. Ren et al. proposed a multi-position self-calibration method for dual-axis RINS [10]. In [11], Zhang et al. analyzed the multi-position calibration method for IMU. In [12], an eight-position self-calibration method for a dual-axis RINS. Syed et al. proposed a multi-position calibration method for MEMS inertial navigation systems [13]. Jiang et al. in [14] have proposed a 36-dimensional KF for systematic calibration. Wen et al. have added gyro-accelerometer asynchronous time as a state variable in self-calibration KF [15]. Jing et al. in [1] have analyzed the data of the gyroscope to improve the accuracy of the calibration. In [16], Song et al. have added inner lever-arm parameters for RINS, which denotes the distance between the sensitive center of the accelerometer and the center of rotation of the IMU. Based on previous research, the authors have derived a complete IMU calibration error model. However, a higher filtering dimension means slower computation and slower convergence. Nowadays, the demand for the rapid response of navigation equipment is higher, and it will be very inappropriate to self-calibration for a few hours before utilization. It should be noted that in high accuracy INS applications (the accuracy of the gyro is better than 0.01 ${}^{\circ }$/h, generally ring laser gyro or fiber optic gyro), the systematic calibration process can last for 3 h.

Hence, many researchers have utilized the backtracking navigation method to shorten the filtering time, this method has been applied in initial alignment widely to shorten the alignment time. Yan et al. proposed a reverse navigation algorithm for INS gyrocompass in-movement alignment [17]. Li has applied the backtracking navigation method in underwater vehicle applications [18]. In [19,20], the authors have proposed a backtracking integration for fast attitude determination-based initial alignment. Li et al. utilized a backtracking navigation scheme for autonomous underwater vehicles [20]. In the application field of RINS, Song et al. proposed a rapid initial alignment scheme for dual-axis RINS [21]. Based on the previous studies, the backtracking navigation scheme has been proved that can effectively shorten the alignment time in the INS initial alignment process. However, there are few studies have applied the backtracking scheme in RINS calibration method.

The backtracking scheme can be applied to the RINS calibration method in theory. However, we need to propose a rotation scheme that can excite all the calibration parameter errors during the rotation process. Hence, a detailed observability analysis is needed. In [15], the authors have analyzed the observability of the 19-position rotation scheme. Tang et al. has utilized piecewise constant systems (PWCS) and singular value decomposition (SVD) method to provide the observability analysis of KF [22]. Cai et al. provided an observability analysis for IMU in a three-axis rotation table [23]. In [24], the authors have studied the observability of strapdown INS alignment. However, the decoupling relationship between the calibration parameters and navigation velocity error has not been studied in previous studies. Therefore, there is a lack of a specific theoretical guide for the rotation path design to excite all the calibration parameter errors.

To solve the above problems, an improved online fast calibration method for dual-axis RINS based on a backtracking scheme is proposed in this paper. The self-calibration method has been studied by many researchers. However, traditional calibration methods need several hours to converge, which is unable to meet the demand for quick response to positioning and orientation. Kalman filtering has both accuracy and real-time performance, but the convergence is slow, so our method can ensure short-term convergence and the real-time performance of the algorithm. The processer we utilize in dual-axis RINS is DSP6748, it has lower computing power because it is a low-power chip. So KF is used in the system. To solve the above problems, we do the following work in this study: (1) we propose a 39-dimensional online calibration Kalman filtering (KF) model to estimate all calibration parameters; (2) Error relationship between calibration parameter errors and navigation error is derived, which can be a theoretical guide for the design of calibration rotation path; (3) A backtracking filtering scheme is proposed to shorten the calibration process. Experimental results indicates that the proposed method can shorten the calibration process to 1 h and improve the calibration accuracy (within 0.5 ppm) simultaneously.

The remainder of this paper is organized as follows: Section 2 gives reference definitions of the proposed calibration method. In Section 3, we derive a 39-dimensional filtering model and analyze the error relationship between calibration parameters error and navigation errors. In Section 4, the online self-calibration method based on the backtracking scheme, is provided. A rotation test is carried out in Section 5 to verify the effectiveness of the proposed method. In Section 6, we give the conclusions.

## 2. Reference Definitions

In the filtering process of the calibration method, the reference definitions are shown as follows:

The earth-centered inertial frame (*i*-frame): *o* locates on the center of the earth, $ox_{i}$ points to the vernal equinox, $oz_{i}$ is the earth’s axis of self-rotation, the inerital information are measured in this reference.

Earth-centred frame (*e*-frame): *o* locates on center of the earth, $ox_{e}$ points to the central meridian, $oz_{e}$ is the earth’s axis of self rotation.

IMU frame (*I*-frame): *o* locates on the rotation center of the IMU, $ox_{I}$, $oy_{I}$ and $oz_{I}$ points to the IMU’s right, forward, and upward, respectively.

vehicle frame (*b*-frame): *o* locates on the rotation center of the vehicle, $ox_{I}$, $oy_{I}$ and $oz_{I}$ points to the vehicle’s right, forward, and upward, respectively.

navigation frame (*n*-frame): The navigation coordinate system is the selected coordinate system when calculating the navigation parameters. In this paper, the east-north-up (E-N-U) geographic coordinate system is used as the navigation coordinate system.

gyro sensitive frame (*g* frame): Non-orthogonal reference frame aligned with gyro-sensitive axes.

accelerometer-sensitive frame (*a* frame): Non-orthogonal reference frame aligned with accelerometer-sensitive axes.

## 3. Sefl-Calibration Filtering Method Design

### 3.1. IMU Error Model

The main measurement components of the IMU are the three-axis gyro component and the three-axis accelerometer component. The input and output models of the gyro and accelerometer can be expressed as [15]:

(1) $$\left\{\begin{array}{c} K_{G}^{-1}N^{g}=T_{g}^{g^{\prime }}\omega_{ig}^{g}+b^{g}+v^{g}\\ K_{A}^{-1}N^{a}=T_{a}^{a^{\prime }}f_{sf}^{a}+f_{A2}^{I}+b^{a}+v^{a} \end{array}\right.$$

where, $K_{G}$ is the scale factor matrix (consists of scale factors of three gyros) of gyroscope, $K_{A}$ is the scale factor matrix of accelerometers, $v^{g}$ is the noise vector of gyros, $v^{a}$ is the noise vector of accelerometers, $\omega_{ig}^{g}$ is the angular velocity sensed by gyros. *g*-frame and *a*-frame denotes the gyroscope and accelerometer’s sensitive axes, *g*-frame and *a*-frame are non-orthogonal references. $N^{g}={\left[\begin{array}{ccc} N_{x}^{g} & N_{y}^{g} & N_{z}^{g} \end{array}\right]}^{T}$ is the primitive output of the gyroscopes, $N^{a}={\left[\begin{array}{ccc} N_{x}^{a} & N_{y}^{a} & N_{z}^{a} \end{array}\right]}^{T}$ is the primitive output of the accelerometers. $\omega_{ig}^{g}$ and $f_{sf}^{a}$ are angular velocity and specific force vector. $K_{G}=diag\left(K_{gx},K_{gy},K_{gz}\right)$ and $K_{A}=diag\left(K_{ax},K_{ay},K_{az}\right)$ are scale factor matrix of the gyroscopes and accelerometers respectivley. $b^{g}={\left[\begin{array}{ccc} K_{G0x} & K_{G0y} & K_{G0z} \end{array}\right]}^{T}$ and $b^{a}={\left[\begin{array}{ccc} K_{A0x} & K_{A0y} & K_{A0z} \end{array}\right]}^{T}$ are the bias vectors of gyroscopes and accelerometers. Accelerometer second-order nonlinear coefficient specific force sensitive term (which can be estimated in high accuracy accelerometers like quartz accelerometer) is described as: $f_{A2}^{I}=M_{f}K_{A2}$, where $M_{f}=diag\left({\left(f_{sfx}^{a}\right)}^{2},{\left(f_{sfy}^{a}\right)}^{2},{\left(f_{sfz}^{a}\right)}^{2}\right)$ is the matrix composed of the square term of the theoretical specific force sensitive to the triaxial accelerometer. $K_{A2}={\left[\begin{array}{ccc} K_{A2x} & K_{A2y} & K_{A2z} \end{array}\right]}^{T}$ is the second-order nonlinear coefficient vector of the accelerometer, it is a part of scale fator.

Write the orthogonal gyro and accelerometer coordinate systems after coordinate frame $g^{\prime }$ and $a^{\prime }$ are compensated by the axis misalignment angle matrix $T_{g}^{g^{\prime }}$ and $T_{a}^{a^{\prime }}$, respectively. The Equation (1) can be rewritten as:

(2) $$\left\{\begin{array}{c} \omega_{ig}^{g}={\left(T_{g}^{g^{\prime }}\right)}^{-1}\left(K_{G}^{-1}N^{g}-b^{g}-v^{g}\right)\\ f_{sf}^{a}={\left(T_{a}^{a^{\prime }}\right)}^{-1}\left(K_{A}^{-1}N^{a}-f_{A2}^{I}-b^{a}-v^{a}\right) \end{array}\right.$$

**Figure 1:**
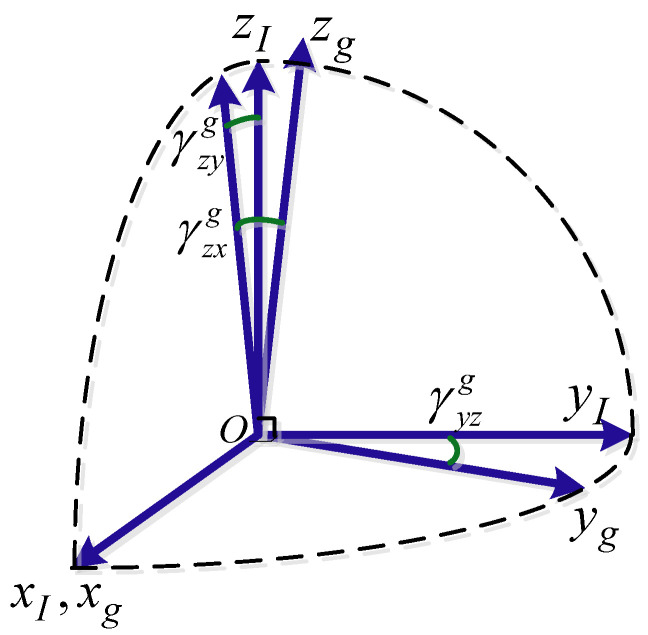
The conversion relationship between the non-orthogonal coordinate frame of the gyro and the orthogonal coordinate frame of the IMU.

As shown in Figure 1, since the measurement information of the gyroscope and accelerometer needs to be projected into the *I* system in the inertia calculation, this paper defines that the measurement center of the gyroscope coincides with the sensitive center of the IMU coordinate system, and the $ox_{I}$ axis of the IMU coordinate system coincides with the sensitive axis $ox_{g}$ of the gyroscope.

Hence, the installation matrix can be written as:

(3) $$T_{g}^{I}\approx \left[\begin{array}{ccc} 1 & 0 & 0\\ -\gamma_{yz}^{g} & 1 & 0\\ \gamma_{zy}^{g} & -\gamma_{zx}^{g} & 1 \end{array}\right]$$

(4) $$T_{a}^{I}=C_{p}^{I}T_{a}^{p}\approx \left[\begin{array}{ccc} 1 & -\eta_{z} & \eta_{y}\\ \eta_{z}-\vartheta_{yz} & 1 & -\eta_{x}\\ -\eta_{y}+\vartheta_{zy} & \eta_{x}-\vartheta_{zx} & 1 \end{array}\right]=\left[\begin{array}{ccc} 1 & \gamma_{xz}^{a} & -\gamma_{xy}^{a}\\ -\gamma_{yz}^{a} & 1 & \gamma_{yx}^{a}\\ \gamma_{zy}^{a} & -\gamma_{zx}^{a} & 1 \end{array}\right]$$

where γ denotes the installation angles, $T_{a}^{p}\approx \left[\begin{array}{ccc} 1 & 0 & 0\\ -\vartheta_{yz} & 1 & 0\\ \vartheta_{zy} & -\vartheta_{zx} & 1 \end{array}\right]$, $\vartheta_{ij}$ is the sensitive non-orthogonal angles from *p*-frame to *a*-frame, *p*-frame is the othogonal reference (The *p*-frame is a transition orthogonal coordinate system), $C_{p}^{I}\approx \left[\begin{array}{ccc} 1 & -\eta_{z} & \eta_{y}\\ \eta_{z} & 1 & -\eta_{x}\\ -\eta_{y} & \eta_{x} & 1 \end{array}\right]$, $\eta_{i}$ is the non-orthogonal angles from *I*-frame to *p*-frame.

Furthermore, the effect of the inner lever arm $r^{I}$ is considered. The inner lever arm refers to the distance vector from the IMU sensitive center (IMU rotation center) to the three-axis accelerometer. When the carrier moves angularly, the inner lever arm will cause the tangential acceleration and centripetal acceleration that the accelerometer is sensitive to. Since the installation error angle of the accelerometer is a small angle, only the inner lever arm in the direction of the sensitive axis of the accelerometer is considered, and the rotational angular acceleration is not considered, the specific force-sensitive term $f_{r}^{I}$ of the inner lever arm of the accelerometer can be expressed as [15]:

(5) $$f_{r}^{I}=M_{\omega }r^{I}$$

where, $M_{\omega }=diag\left[{\left(\omega_{iIy}^{I}\right)}^{2}+{\left(\omega_{iIz}^{I}\right)}^{2},{\left(\omega_{iIx}^{I}\right)}^{2}+{\left(\omega_{iIz}^{I}\right)}^{2},{\left(\omega_{iIx}^{I}\right)}^{2}+{\left(\omega_{iIy}^{I}\right)}^{2}\right]$, $r^{I}={\left[\begin{array}{ccc} r_{x}^{I} & r_{y}^{I} & r_{z}^{I} \end{array}\right]}^{T}$.

Ignoring the noise term in Equation (1), the calibration models of the gyro and accelerometer components in the IMU coordinate system can be expressed as:

(6) $$\left\{\begin{array}{c} \omega_{iI}^{I}=K_{G}^{-1}N^{g}-\omega_{0}^{I}\\ f_{sf}^{I}=K_{A}^{-1}N^{a}-f_{A2}^{I}-f_{0}^{I} \end{array}\right.$$

The error equation is the differential equation of the above equations, based on Equations (2) and (6). $\omega_{0}^{I}$ is the gyro bias vector, $f_{0}^{I}$ is the accelerometer bias vector.

### 3.2. 39-Dimensional Kalman Filtering Model

The error parameters of the system-level filter self-calibration include two categories. The first category is the various error parameters involved in the IMU measurement error model; the second category is the observation error parameters represented by the outer lever arm error. The outer lever arm error refers to the sensitivity of the IMU. The distance error vector from the center to the center of the turntable will stimulate the observation velocity error and the observation position error when the IMU rotates [15]. The outter lever arm are written as $\delta l^{I}$, The relationship between the corresponding true value and the measured value can be expressed as: $l^{I}={\tilde{l}}^{I}-\delta l^{I}$. Bsed on the previous analysis, combined with the inertial navigation error equation, attitude misalignment angle error $\phi^{n}={\left[\begin{array}{ccc} \phi_{E}^{n} & \phi_{N}^{n} & \phi_{U}^{n} \end{array}\right]}^{T}$, the velocity error $\delta v_{}^{n}={\left[\begin{array}{ccc} \delta v_{E}^{n} & \delta v_{N}^{n} & \delta v_{U}^{n} \end{array}\right]}^{T}$ and the position error $\delta p={\left[\begin{array}{ccc} \delta L & \delta \lambda & \delta h \end{array}\right]}^{T}$ into the state vector into consideration, the state variable of the KF can be written as:

(7) $$X_{C}={\left[\begin{array}{cccccc} {\left(\phi^{n}\right)}^{T} & {\left(\delta v_{}^{n}\right)}^{T} & {\left(\delta p\right)}^{T} & {\left(X_{g}\right)}^{T} & {\left(X_{a}\right)}^{T} & {\left(\delta l^{I}\right)}^{T} \end{array}\right]}^{T}$$

where, $X_{g}={\left[\begin{array}{cccccc} \delta k_{11}^{g} & \delta k_{21}^{g} & \delta k_{31}^{g} & \delta k_{22}^{g} & \delta k_{32}^{g} & \delta k_{33}^{g} \end{array}\begin{array}{ccc} \varepsilon_{x} & \varepsilon_{y} & \varepsilon_{z} \end{array}\right]}^{T}$.

$\begin{array}{c} X_{a}=[\begin{array}{cccccc} \delta k_{11}^{a} & \delta k_{21}^{a} & \delta k_{31}^{a} & \delta k_{12}^{a} & \delta k_{22}^{a} & \delta k_{32}^{a} \end{array}\\ \begin{array}{ccccccccc} \delta k_{13}^{a} & \delta k_{23}^{a} & \delta k_{33}^{a} & \nabla_{x} & \nabla_{y} & \nabla_{z} & \delta KT_{x}^{a} & \delta KT_{y}^{a} & \delta KT_{z}^{a} \end{array}{]}^{T} \end{array}$. $\delta k_{ij}^{g}$ is the element of $K_{G}^{-1}$, $\varepsilon_{i}$ ($\omega_{0}^{I}$) is the gyro bias, $\delta k_{ij}^{a}$ is the element of $K_{A}^{-1}$, and $\nabla_{x}$ ($f_{0}^{I}$) is the accelerometer bias.

The state transform funciton can be written as:

(8) $${\dot{X}}_{C}=F_{C}X_{C}+G_{C}W_{C}$$

where, $F_{C}$ represents the state transition matrix, $G_{C}$ represents the system noise driving matrix, $W_{C}$ denotes the system nosie matrix, $\varepsilon_{w}^{I}={\left[\begin{array}{ccc} \varepsilon_{wx}^{I} & \varepsilon_{wy}^{I} & \varepsilon_{wz}^{I} \end{array}\right]}^{T}$ and $\nabla_{w}^{I}={\left[\begin{array}{ccc} \nabla_{wx}^{I} & \nabla_{wy}^{I} & \nabla_{wz}^{I} \end{array}\right]}^{T}$ are the random noise matrix of gyroscopes and accelerometers. $F_{C}$ amd $G_{C}$ can be written as:

(9) $$F_{C}=\left[\begin{array}{c} \begin{array}{cccccc} -\left(\omega_{in}^{n}\times \right) & F_{12} & F_{13} & F_{14} & 0_{3\times 18} & 0_{3\times 3}\\ \left(f_{sf}^{n}\times \right) & F_{22} & F_{23} & 0_{3\times 9} & F_{25} & 0_{3\times 3}\\ 0_{3\times 3} & F_{32} & F_{33} & 0_{3\times 9} & 0_{3\times 18} & 0_{3\times 3} \end{array}\\ 0_{30\times 39} \end{array}\right]$$

(10) $$G_{C}=\left[\begin{array}{c} \begin{array}{cc} -C_{I}^{n} & 0_{3\times 3}\\ 0_{3\times 3} & C_{I}^{n} \end{array}\\ 0_{33\times 6} \end{array}\right]$$

where, $C_{I}^{n}$ is the transform matrix from *I*-frame to *n*-frame. $\omega_{ie}^{}$ represents the earth self-rotatoin angular velocity. $\omega_{in}^{n}$ is the angular velocity of *n*-frame to *i*-frame. *L* is the latitude. $R_{M}$ denotes the radius of curvature of the meridian circle, $R_{N}$ represents the the radius of curvature of the unitary circle, *h* is the height of the IMU. The elements of the $F_{C}$ are shown as: $F_{12}=M_{2}$, $F_{13}=M_{1}+M_{3}$, $F_{14}=-C_{I}^{n}M_{g}$, $F_{22}=-\left[\left(v^{n}\times \right)F_{12}+\left(2\omega_{ie}^{n}+\omega_{en}^{n}\right)\times \right]$, $M_{1}=\left[\begin{array}{ccc} 0 & 0 & 0\\ -\omega_{ie}\sin L & 0 & 0\\ \omega_{ie}\cos L & 0 & 0 \end{array}\right]$,

$$M_{2}=\left[\begin{array}{ccc} 0 & -\frac{1}{R_{M}+h} & 0\\ \frac{1}{R_{N}+h} & 0 & 0\\ \frac{\tan L}{R_{N}+h} & 0 & 0 \end{array}\right],M_{3}=\left[\begin{array}{ccc} 0 & 0 & \frac{v_{N}}{{\left(R_{N}+h\right)}^{2}}\\ 0 & 0 & -\frac{v_{E}}{{\left(R_{N}+h\right)}^{2}}\\ \frac{v_{E}\sec^{2}L}{R_{N}+h} & 0 & -\frac{v_{E}\tan L}{{\left(R_{N}+h\right)}^{2}} \end{array}\right],$$

$$F_{23}=\left(v^{n}\times \right)\left(2M_{1}+M_{3}\right),$$

$$F_{25}=C_{I}^{n}M_{a},F_{32}=\left[\begin{array}{ccc} 0 & \frac{1}{R_{M}+h} & 0\\ \frac{1}{(R_{N}+h)\cos L} & 0 & 0\\ 0 & 0 & 1 \end{array}\right],F_{33}=\left[\begin{array}{ccc} 0 & 0 & -\frac{v_{N}}{{\left(R_{N}+h\right)}^{2}}\\ \frac{v_{E}\sec L\tan L}{R_{N}+h} & 0 & -\frac{v_{E}\sec L}{{\left(R_{N}+h\right)}^{2}}\\ 0 & 0 & 0 \end{array}\right].$$

$$M_{g}=\left[\begin{array}{cccc} {\tilde{\omega }}_{ibx}^{I}I_{3\times 3} & \left[\begin{array}{c} 0_{1\times 2}\\ {\tilde{\omega }}_{iIy}^{I}I_{2\times 2} \end{array}\right] & \left[\begin{array}{c} 0_{2\times 1}\\ {\tilde{\omega }}_{iIz}^{I} \end{array}\right] & I_{3\times 3} \end{array}\right],$$

$$M_{a}=\left[\begin{array}{cccccc} {\tilde{f}}_{sfx}^{I}I_{3\times 3} & {\tilde{f}}_{sfy}^{I}I_{3\times 3} & {\tilde{f}}_{sfz}^{I}I_{3\times 3} & {\tilde{M}}_{f2} & {\tilde{M}}_{\omega } & I_{3\times 3} \end{array}\right],\mathrm{where},$$

$${\tilde{M}}_{f}=diag\left[{\left({\tilde{f}}_{sfx}^{I}\right)}^{2},{\left({\tilde{f}}_{sfy}^{I}\right)}^{2},{\left({\tilde{f}}_{sfz}^{I}\right)}^{2}\right],$$

$${\tilde{M}}_{\omega }=diag\left[{\left({\tilde{\omega }}_{iIy}^{I}\right)}^{2}+{\left({\tilde{\omega }}_{iIz}^{I}\right)}^{2},{\left({\tilde{\omega }}_{iIx}^{I}\right)}^{2}+{\left({\tilde{\omega }}_{iIz}^{I}\right)}^{2},{\left({\tilde{\omega }}_{iIx}^{I}\right)}^{2}+{\left({\tilde{\omega }}_{iIy}^{I}\right)}^{2}\right].$$

To establish the measurement equation of the dual-axis RINS system-level self-calibration filtering method, the measurement information should be determined first. The velocity and position measurement equation is expressed as follows:

(11) $$\left\{\begin{array}{c} {\tilde{v}}_{mea}^{n}={\tilde{v}}_{}^{n}+{\tilde{C}}_{I}^{n}\left[\left({\tilde{\omega }}_{iI}^{I}-{\tilde{C}}_{n}^{I}{\tilde{\omega }}_{ie}^{n}\right)\times {\tilde{l}}^{I}\right]\\ {\tilde{p}}_{mea}^{n}=\tilde{p}+{\tilde{F}}_{32}{\tilde{C}}_{I}^{n}{\tilde{l}}^{I} \end{array}\right.$$

where, ${\tilde{C}}_{I}^{n}=\left[I_{3\times 3}-\left(\phi^{n}\times \right)\right]C_{I}^{n}$ represents the calculated attitude matrix with attitude misalignment.

The measurement transform equation can be written as:

(12) $$\begin{array}{c} Z_{C}^{n}=\left[\begin{array}{c} Z_{Cv}^{n}\\ Z_{Cp}^{n} \end{array}\right]=\left[\begin{array}{c} {\tilde{v}}_{mea}^{n}-v_{obv}^{n}\\ {\tilde{p}}_{mea}-p_{obv} \end{array}\right]\\ =H_{C}X_{C}+V_{C} \end{array}$$

where $Z_{C}^{n}$ denotes the measurement vector, $H_{C}$ is the measurement matrix,

(13) $$H_{C}=\left[\begin{array}{cccccc} H_{11} & I_{3\times 3} & H_{13} & H_{14} & 0_{3\times 18} & H_{16}\\ H_{21} & 0_{3\times 3} & H_{23} & 0_{3\times 9} & 0_{3\times 18} & H_{26} \end{array}\right]$$

where $H_{21}=F_{32}\left[\left(C_{I}^{n}l^{I}\right)\times \right]$, $H_{23}=\left[\begin{array}{ccc} 1 & 0 & -\frac{{\left(C_{I}^{n}l^{I}\right)}_{y}}{{\left(R_{M}+h\right)}^{2}}\\ \frac{\sin L{\left(C_{I}^{n}l^{I}\right)}_{x}}{\left(R_{N}+h\right)\cos^{2}L} & 1 & -\frac{{\left(C_{I}^{n}l^{I}\right)}_{x}}{{\left(R_{N}+h\right)}^{2}\cos L}\\ 0 & 0 & 1 \end{array}\right]$, $H_{26}=F_{32}C_{I}^{n}$.

So far, the derivation of the 39-dimensional filtering equation is completed.

### 3.3. Self-Calibration Parameter Error Excitation and Coupling Analysis

To carry out the coupling anaysis of each calibration parameters, the error propagation law of each self-calibration parameters are derived first. Assuming that the initial state *b*-frame and *n*-frame are coincide ($C_{b}^{n}\left(t\right)=I_{3}$), considering the case where the *y*-axis of the IMU continues to rotate in the north direction at time period $\left[0,T\right]$ with an angular velocity $\omega_{r}\left(\omega_{r}\gg \omega_{ie}\right)$. The measured angular velocity can be expressed as: ${\tilde{\omega }}_{iI}^{I}\approx {\left[\begin{array}{ccc} 0 & \omega_{r} & 0 \end{array}\right]}^{T}+\delta \omega_{iI}^{I}$. The measured specific force can be expressed as ${\tilde{f}}_{sf}^{I}={\left[\begin{array}{ccc} -g\sin\left(\omega_{r}T\right) & 0 & g\cos\left(\omega_{r}T\right) \end{array}\right]}^{T}+\delta f_{sf}^{I}$, the attitude transform matrix can be described as $C_{I}^{n}\left(t\right)=C_{b}^{n}\left(t\right)C_{I}^{b}\left(t\right)$. Ignoring higher-order episilon (more than two-order), The measurement error due to the $\delta K_{G}$ and bias $\varepsilon^{I}$ can be described as:

(14) $$\begin{array}{c} \delta {\dot{Z}}_{Cv}^{n}\left(\delta K_{G},\varepsilon^{I}\right)\approx \left(g^{n}\times \right)\int_{0}^{T}C_{b}^{n}\left(t\right)C_{I}^{b}\left(t\right)M_{g}\left(t\right)dt\cdot X_{g}\\ \approx \left(g^{n}\times \right)\int_{0}^{T}\left(\left[\begin{array}{ccc} 1 & 0 & 0\\ 0 & 1 & 0\\ 0 & 0 & 1 \end{array}\right]\left[\begin{array}{ccc} \cos\left(\omega_{r}T\right) & 0 & \sin\left(\omega_{r}T\right)\\ 0 & 1 & 0\\ -\sin\left(\omega_{r}T\right) & 0 & \cos\left(\omega_{r}T\right) \end{array}\right]\right)dt\cdot \left[\begin{array}{c} \varepsilon_{x}^{I}\\ \omega_{r}\delta k_{yy}^{g}+\varepsilon_{y}^{I}\\ \omega_{r}\delta k_{zy}^{g}+\varepsilon_{z}^{I} \end{array}\right]\\ =\left[\begin{array}{ccc} 0 & g & 0\\ -g & 0 & 0\\ 0 & 0 & 0 \end{array}\right]\left[\begin{array}{ccc} \frac{\sin\left(\omega_{r}T\right)}{\omega_{r}} & 0 & -\frac{\cos\left(\omega_{r}T\right)-1}{\omega_{r}}\\ 0 & T & 0\\ \frac{\cos\left(\omega_{r}T\right)-1}{\omega_{r}} & 0 & \frac{\sin\left(\omega_{r}T\right)}{\omega_{r}} \end{array}\right]\left[\begin{array}{c} \varepsilon_{x}^{I}\\ \omega_{r}\delta k_{yy}^{g}+\varepsilon_{y}^{I}\\ \omega_{r}\delta k_{zy}^{g}+\varepsilon_{z}^{I} \end{array}\right]\\ =\left[\begin{array}{c} g\omega_{r}T\delta k_{yy}^{g}+gT\varepsilon_{y}^{I}\\ g\cos\left(\omega_{r}T\right)\left(\delta k_{zy}^{g}+\frac{\varepsilon_{z}^{I}}{\omega_{r}}\right)-\frac{g\sin\left(\omega_{r}T\right)\varepsilon_{x}^{I}}{\omega_{r}}-g\delta k_{zy}^{g}-\frac{g\varepsilon_{z}^{I}}{\omega_{r}}\\ 0 \end{array}\right] \end{array}$$

Ignoring higher-order epsilon, The measurement error due to the $\delta K_{A}$ and $\nabla^{I}$ can be described as:

(15) $$\begin{array}{c} \delta {\dot{Z}}_{Cv}^{n}\left(\delta K_{A},\nabla^{I}\right)=C_{I}^{n}M_{a}\left(:,1:12\right)X_{a}\left(:,1:12\right)\\ \approx \left[\begin{array}{ccc} \cos\left(\omega_{r}T\right) & 0 & \sin\left(\omega_{r}T\right)\\ 0 & 1 & 0\\ -\sin\left(\omega_{r}T\right) & 0 & \cos\left(\omega_{r}T\right) \end{array}\right]\left[\begin{array}{c} -g\sin\left(\omega_{r}T\right)\delta k_{xx}^{a}+g\cos\left(\omega_{r}T\right)\delta k_{xz}^{a}+\nabla_{x}^{b}\\ -g\sin\left(\omega_{r}T\right)\delta k_{yx}^{a}+g\cos\left(\omega_{r}T\right)\delta k_{yz}^{a}+\nabla_{y}^{b}\\ -g\sin\left(\omega_{r}T\right)\delta k_{zx}^{a}+g\cos\left(\omega_{r}T\right)\delta k_{zz}^{a}+\nabla_{z}^{b} \end{array}\right]\\ =\left[\begin{array}{c} \begin{array}{c} \frac{g\sin\left(2\omega_{r}T\right)}{2}\left(\delta k_{zz}^{a}-\delta k_{xx}^{a}\right)+\frac{g}{2}\left(\delta k_{xz}^{a}-\delta k_{zx}^{a}\right)+\frac{g\cos\left(2\omega_{r}T\right)}{2}\left(\delta k_{xz}^{a}+\delta k_{zx}^{a}\right)+\\ \nabla_{x}^{b}\cos\left(\omega_{r}T\right)+\nabla_{z}^{b}\sin\left(\omega_{r}T\right) \end{array}\\ -g\sin\left(\omega_{r}T\right)\delta k_{yx}^{a}+g\cos\left(\omega_{r}T\right)\delta k_{yz}^{a}+\nabla_{y}^{b}\\ \begin{array}{c} \frac{g}{2}\left(\delta k_{xx}^{a}+\delta k_{zz}^{a}\right)+\frac{g\cos\left(2\omega_{r}T\right)}{2}\left(\delta k_{zz}^{a}-\delta k_{xx}^{a}\right)-\frac{g\sin\left(2\omega_{r}T\right)}{2}\left(\delta k_{xz}^{a}+\delta k_{zx}^{a}\right)-\\ \nabla_{x}^{b}\sin\left(\omega_{r}T\right)+\nabla_{z}^{b}\cos\left(\omega_{r}T\right) \end{array} \end{array}\right] \end{array}$$

Similarly, the measurement error due to the $\delta K_{A2}$ can be described as:

(16) $$\begin{array}{c} \delta {\dot{Z}}_{Cv}^{n}\left(\delta K_{A2}\right)=C_{I}^{n}{\tilde{M}}_{f2}X_{a}\left(:,13:15\right)\\ \approx \left[\begin{array}{ccc} \cos\left(\omega_{r}T\right) & 0 & \sin\left(\omega_{r}T\right)\\ 0 & 1 & 0\\ -\sin\left(\omega_{r}T\right) & 0 & \cos\left(\omega_{r}T\right) \end{array}\right]\left[\begin{array}{c} g^{2}\sin^{2}\left(\omega_{r}T\right)\delta K_{Ax2}\\ 0\\ g^{2}\cos^{2}\left(\omega_{r}T\right)\delta K_{Az2} \end{array}\right]\\ =\left[\begin{array}{c} g^{2}\sin\left(2\omega_{r}t\right)\left(\sin\left(\omega_{r}t\right)\delta K_{Ax2}+\cos\left(\omega_{r}t\right)\delta K_{Az2}\right)/2\\ 0\\ -g^{2}\sin^{3}\left(\omega_{r}t\right)\delta K_{Ax2}+g^{2}\cos^{3}\left(\omega_{r}t\right)\delta K_{Az2} \end{array}\right] \end{array}$$

The measurement error due to the $\delta r^{I}$ can be written as:

(17) $$\begin{array}{c} \delta {\dot{Z}}_{v}^{n}\left(\delta r^{I}\right)=C_{I}^{n}{\tilde{M}}_{\omega }X_{a}\left(:,16:18\right)\\ \approx \left[\begin{array}{ccc} \cos\left(\omega_{r}T\right) & 0 & \sin\left(\omega_{r}T\right)\\ 0 & 1 & 0\\ -\sin\left(\omega_{r}T\right) & 0 & \cos\left(\omega_{r}T\right) \end{array}\right]\left[\begin{array}{c} {\left(\omega_{r}\right)}^{2}\delta r_{x}^{I}\\ 0\\ {\left(\omega_{r}\right)}^{2}\delta r_{z}^{I} \end{array}\right]\\ =\left[\begin{array}{c} {\left(\omega_{r}\right)}^{2}\left(\cos\left(\omega_{r}T\right)\delta r_{x}^{I}+\sin\left(\omega_{r}T\right)\delta r_{z}^{I}\right)\\ 0\\ {\left(\omega_{r}\right)}^{2}\left(-\sin\left(\omega_{r}T\right)\delta r_{x}^{I}+\cos\left(\omega_{r}T\right)\delta r_{z}^{I}\right) \end{array}\right] \end{array}$$

Based on Equations (14)–(17), The error propagation form of self-calibration parameters under transposition excitation are summarized in Table 1:

Based on Table 1, the error propagation forms of the $\delta k_{zy}^{g}$ and $\delta k_{yz}^{g}$, $\delta k_{xz}^{a}$ and $\delta k_{zx}^{a}$ are the same respectively. When the IMU rotates along the *y*-axis, the above two sets of installation error parameters will always have a coupling relationship. Similarly, the IMU rotates along the *x*-axis, The error propagation forms of the three groups of parameters $\delta k_{xy}^{a}$ and $\delta k_{yx}^{g}$, $\delta k_{xz}^{a}$ and $\delta k_{zx}^{g}$, $\delta k_{zy}^{a}$ and $\delta k_{yz}^{a}$ are respectively the same. When the IMU rotates along the *z*-axis, the error propagation forms of the $\delta k_{yx}^{a}$ and $\delta k_{xy}^{a}$ are the same.

Although there are 1–3 sets of coupling relationships in the installation errors of the gyro and the accelerometer during the horizontal rotation of the three sensitive axes of the IMU, such coupling relationships can be decoupled in turn through a specific indexing method.

Except for the coupling term in the installation error, there is no error term in the same propagation form during the horizontal rotation of the three sensitive axes of the IMU. According to the error propagation form of each system-level self-calibration parameter, the longest error propagation period is $2\pi /\omega_{r}$ (derived by Equation (17)).

Based on the previous analysis, the rotation path of the self-calibration can be designed as Table 2 [15]:

The angular rotation rate is set as 20 ${}^{\circ }$/s (the angular rate is not unique, usually 5 ${}^{\circ }$/s to 20 ${}^{\circ }$/s are commonly used), after finishing the last rotation, stay static until 1800 s to end the calibration process.

In [15] the error parameters errors of IMU scale factors and biases are proved observable. Therefore, we need to analyze the observability of inner lever arms and accelerometer second-order factors in this study. Using the PWCS and SVD methods, the observability using the designed excitation path is shown as follows in Table 3.

## 4. Self-Calibration Process Based on Backtracking Scheme

The reverse navigation algorithm is a time-reversed algorithm based on the forward navigation algorithm. It is usually used to prolong the filtering time so that the filter can complete the convergence in a relatively short time. In the process of reverse navigation, the speed of the solution needs to be reversed. The reverse navigation algorithm can be rewritten as the following:

(18) $$\begin{array}{c} C_{bk-1}^{n}=C_{bk}^{n}\left(I_{3}+T_{s}{\tilde{\Omega }}_{nbk}^{b}\right)\\ -v_{k-1}^{n}=-v_{k}^{n}+T_{s}a_{nbk-1}^{n} \end{array}$$

where, $\Omega_{nbk}^{b}=\left(\omega_{nbk}^{b}\times \right)$.

The detailed derivation of reverse navigation method has been fully explained in reference [17], hence, reverse navigation process can be summarized as:

(19) $$\begin{array}{c} {\overset{\leftarrow }{C}}_{bp}^{n}={\overset{\leftarrow }{C}}_{bp-1}^{n}(I+T_{s}{\overset{\leftarrow }{\Omega }}_{nbp}^{b})\\ {\overset{\leftarrow }{v}}_{p}^{n}={\overset{\leftarrow }{v}}_{p-1}^{n}+T_{s}{\overset{\leftarrow }{a}}_{p-1,p}\\ {\overset{\leftarrow }{L}}_{p}={\overset{\leftarrow }{L}}_{p-1}+T_{s}{\overset{\leftarrow }{v}}_{Np-1}^{n}/(R_{M}+{\overset{\leftarrow }{h}}_{p-1})\\ {\overset{\leftarrow }{\lambda }}_{p}={\overset{\leftarrow }{\lambda }}_{p-1}+T_{s}v_{Ep-1}^{n}\sec{\overset{\leftarrow }{L}}_{p-1}/(R_{N}+{\overset{\leftarrow }{h}}_{p-1})\\ {\overset{\leftarrow }{h}}_{p}={\overset{\leftarrow }{h}}_{p-1}+T_{s}{\overset{\leftarrow }{v}}_{Up-1}^{n} \end{array}$$

where, p=m−k+1.

As shown in Equation (19), the algorithm form of the reverse navigation algorithm is similar to that of the forward navigation. It only needs to invert the speed, the angular rate of the earth’s rotation, and the stored gyro output.

In the process of reverse navigation, due to the minus output of the gyroscope, the output of the gyroscope can be written as:

(20) $$F_{14}=-C_{b}^{n}\left[\begin{array}{cccc} N_{x}^{g}I_{3\times 3} & \left[\begin{array}{c} 0_{1\times 2}\\ N_{y}^{g}I_{2\times 2} \end{array}\right] & \left[\begin{array}{c} 0_{2\times 1}\\ N_{z}^{g} \end{array}\right] & -I_{3\times 3} \end{array}\right]$$

Other filter variables are consistent with the forward self-calibration filter model.

**Figure 2:**
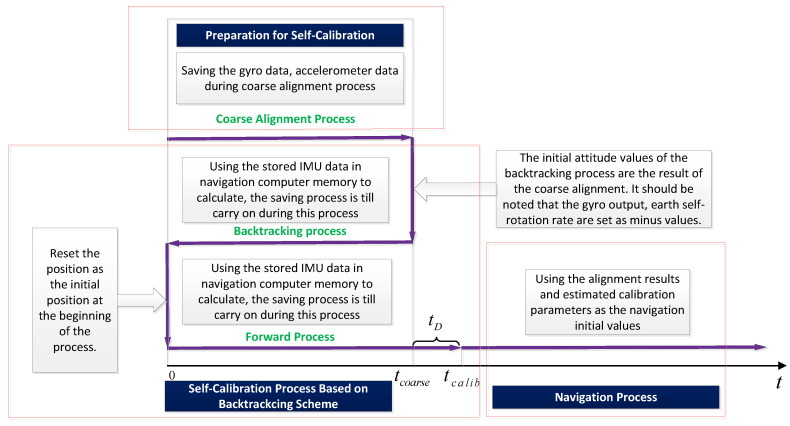
Diagram of self-calibration process based on backtracking scheme.

To complete the self-calibration process of the dual-axis RINS in a short time, it is necessary to propose a method to enable the self-calibration filter to be able to convergence is accomplished rapidly. Therefore, this paper proposes a fast self-calibration algorithm architecture based on a backtracking scheme, the architecture is shown in Figure 2 as follows:

The 39D KF method is to decouple the error through the specified indexing sequence, to decouple the relationship between the navigation error and the IMU error, and use Kalman filtering to estimate. To ensure the linearity of the KF, we usually set initial calibration parameters at the beginning of the filtering process. The self-calibration filtering process-based backtracking scheme proposed in this paper makes full use of IMU information. Under the condition that the initial calibration parameters are set (inaccurate) (the coarse alignment is necessary, providing a coarse attitude can guarantee the linearity of the Kaman filter), the RINS can obtain an initial attitude after coarse alignment. The raw data of the IMU is stored at the same time. Then, utilize the attitude value after coarse alignment as the initial attitude value, utilize the stored gyroscope and accelerometer data to perform the reverse self-calibration process, and continue to store the IMU data. After the reverse filtering process end, the forward filtering process is performed from the initial moment, because the calculation rate of the self-calibration filtering process is faster than the rate of storing the data, and the final forward filtering process will end at time $t_{calib}$, which can catch up with the data stored in real-time and finally enter into the navigation process.

Given the above explanations, the self-calibration based on the backtracking scheme can be summarized as follows:

1. The information from the accelerometer and gyroscope are stored in the memory in real-time during the time period from the start (time 0) to the end (time $t_{coarse}$) of the coarse alignment stage of the dual-axis RINS, and the coarse alignment process ends at time $t_{coarse}$. We obtain attitude information with an acceptable error.
2. The process of reverse self-calibration filtering starts at the end of the coarse alignment of the dual-axis RINS (time $t_{coarse}$), and the process of reverse self-calibration filtering towards the start of the coarse alignment stage (time 0), using the stored gyro data, the angular velocity of the earth’s rotation, and the reverse self-calibration The velocity at the initial moment of filtering needs to be negated. At the same time, in the process of reverse self-calibration filtering, the information of the gyroscope and accelerometer are still stored in the memory of the navigation computer.
3. After the reverse self-calibration filtering process ends, the program executes to the start time of the coarse alignment stage (time 0), and from this time onwards, the forward self-calibration filtering process is performed without modifying all the parameters of the Kalman filter used in the previous stage, the velocity at time 0 calculated using the reverse self-calibration filter needs to be reversed. At the same time, the stored gyro information and the Earth’s rotation angular rate also need to be returned to the normal state from the previous inversion state (no need to invert). Since the calculation rate of the navigation computer is not fast enough to be ignored, the forward self-calibration filtering process needs to catch up with the stored time $t_{calib}$ until.
4. The above three steps are the implementation process of the fast self-calibration algorithm based on backtracking navigation. At present, most navigation computers are equipped with large-capacity storage elements such as DDR2, SD card, etc., so that the online fast self-calibration algorithm of dual-axis RINS can be realized.

It should be noted that if the filter still does not fully converge after one round of retrospective filtering calibration, the next round of retrospective filtering and calibration can be performed. The reverse filtering process starts from the previous $t_{calib}$, and the subsequent process is the same as before.

## 5. Experimental Results and Analysis

**Figure 3:**
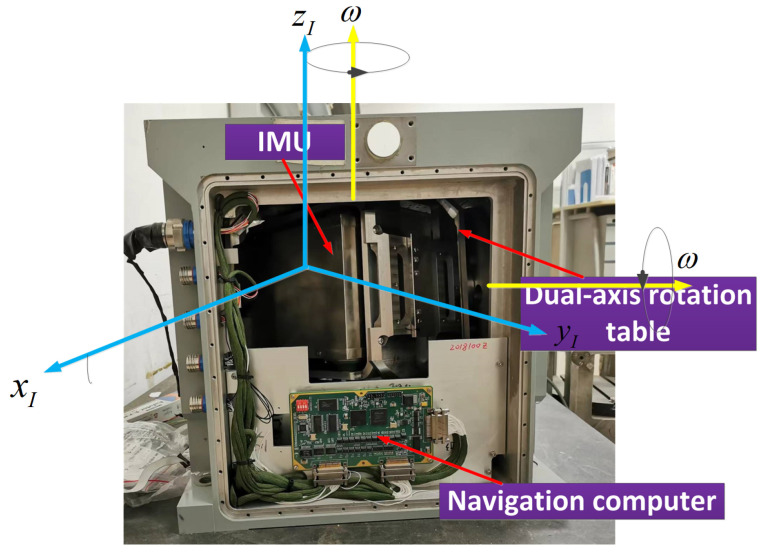
Dual-axis RINS.

To verify the effectiveness of the proposed self-calibration method based on the backtracking scheme, we conduct a static test to evaluate the accuracy of the calibration parameters. The dual-axis we utilize in this study is shown in Figure 3. The dual-axis RINS realize the dual-axis turntable with 3D-IMU.

**Figure 4:**
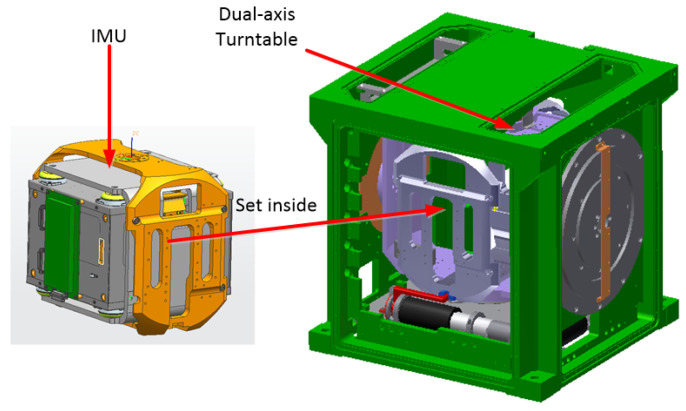
Structure of the Dual-axis RINS.

The structure of the dual-axis RINS is shown as follows Figure 4, and the IMU is installed inside the dual-axis turntable to realize the self-contained dual-axis RINS.

**Figure 5:**
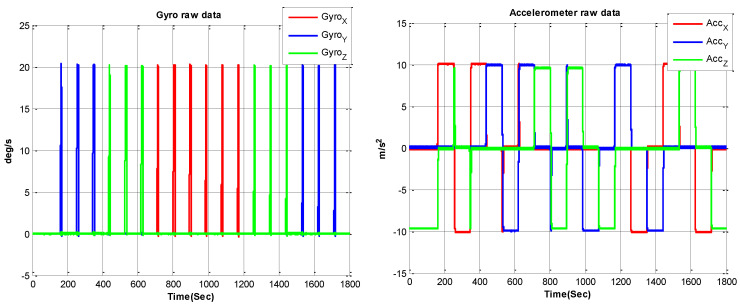
IMU raw data.

The gyroscope we use in the RINS is a laser ring gyro (RLG), the accuracy of the RLG is 0.003 ${}^{\circ }$/h (100 s, 1σ), with a 1 ppm (1σ) of scale factor repeatability. The quartz accelerometers have an accuracy of 20 μg (1σ). The sampling frequency of the IMU is 1000 Hz. To show the calibration process more clearly, the raw plot of the IMU is shown in Figure 5.

**Figure 6:**
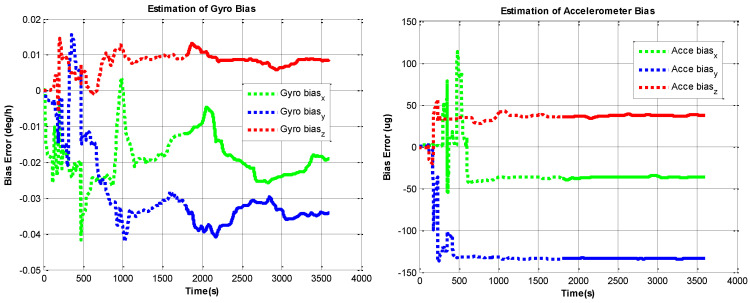
Estimation curves of gyroscope and accelerometer bias errors.

**Figure 7:**
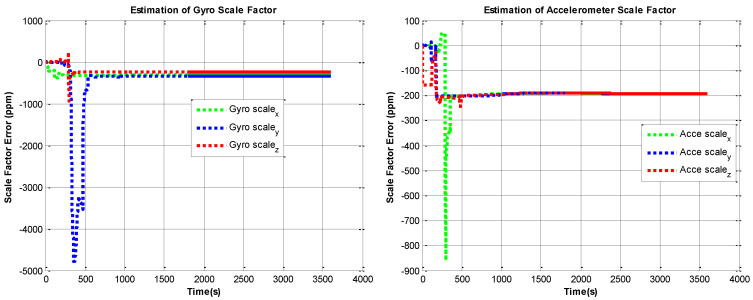
Estimation curves of gyroscope and accelerometer scale factor errros.

**Figure 8:**
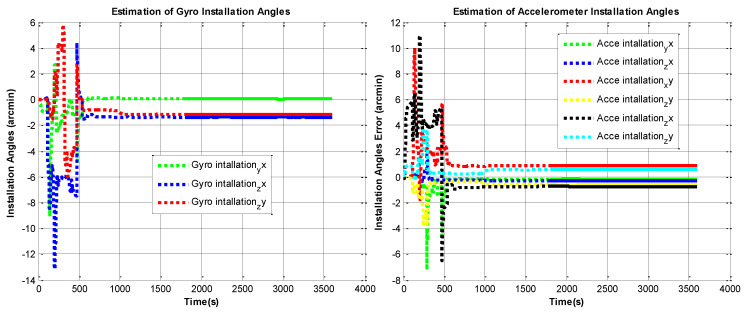
Estimation curves of gyroscope and accelerometer installation angle errors.

**Figure 9:**
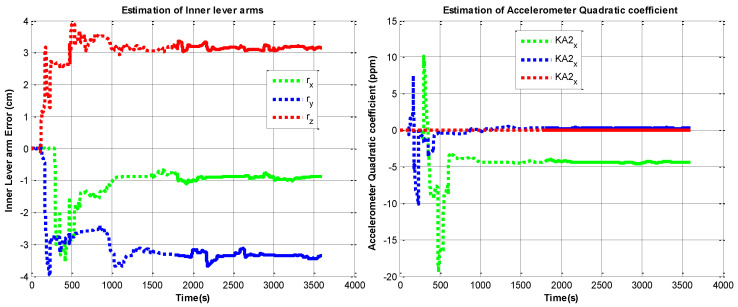
Estimation curves of inner lever arm and accelerometer quadratic coefficient errors.

The RINS is fixed in a marble, the algorithm is implemented on the digital signal processer (DSP) chip. We use the method in reference to [10] as a comparison. The self-calibration process lasts 30 mins (the backtracking process also lasts 30 min, with the same data). We use a high-accuracy three-axis turntable to calibrate the IMU parameters as reference [25], this method requires a high-accuracy turntable, the IMU needs to be removed from the dual-axis RINS, as the accuracy of the dual-axis turntable is not high enough (especially horizontal accuracy). The traditional method is described in [15]. The estimation curves of the IMU parameters are shown in Figure 6, Figure 7, Figure 8 and Figure 9, the dotted line part is the reverse filtering process, and the solid line part is the forward filtering process. We can find out all parameters are converged after the backtracking scheme ended.

To verify the estimation accuracy of the parameters, the estimated parameters can be summarized as Table 4.

As shown in Table 4, the estimation accuracy of the proposed method is better than the traditional method, especially the calibration parameters of gyros. The errors of gyro biases estimated by the traditional method are 0.02 ${}^{\circ }$/h to 0.03 ${}^{\circ }$/h, using the proposed method, the errors are only within 0.005 ${}^{\circ }$/h. The errors of the gyro scale factors estimated by the traditional method are more than 4 ppm.

Compared with the traditional self-calibration process, in a relatively short time, the method proposed in this paper achieves high estimation accuracy, which can verify the effectiveness of the proposed fast self-calibration method based on the backtracking scheme.

## 6. Conclusions

In the field of the rotational inertial navigation system, many fields (land and aircraft) have put forward “three-self” performance requirements, namely self-checking, self-alignment, and self-calibration. Although the traditional strapdown INS can realize self-checking and self-alignment, it cannot realize self-calibration. In response to such problems, dual-axis came into research, and the introduction of the dual-axis indexing mechanism fundamentally solved the problem of self-calibration without disassembly of IMU from the dual-axis turntable. Therefore, dual-axis RINS has received extensive attention in the field of land navigation. The accuracy of the calibration parameters can determine the navigation accuracy of RINS. The traditional self-calibration method needs several hours to converge. To shorten the self-calibration time by more than 50 percent, we propose a 39-dimensional online calibration Kalman filtering (KF) model to estimate all calibration parameters. Error relationship between calibration parameter errors and navigation errors are derived, which can be a theoretical guide for the design of calibration rotation path. A backtracking filtering scheme is proposed to shorten the calibration process. Experimental results indicate that the proposed method can shorten the calibration process and improve the calibration accuracy simultaneously compared with the traditional self-calibration method.

## Figures and Tables

**Table 1:** Error propagation form of self-calibration parameters under transposition excitation.

Parameters	$\delta {\dot{Z}}_{Cv_{E}}^{n}$	$\delta {\dot{Z}}_{Cv_{N}}^{n}$	$\delta {\dot{Z}}_{Cv_{U}}^{n}$
$\delta k_{zy}^{g}$	–	$g\cos\left(\omega_{r}T\right)$	–
$\delta k_{yz}^{a}$	–	$g\cos\left(\omega_{r}T\right)$	–
$\delta k_{yx}^{a}$	–	$-g\sin\left(\omega_{r}T\right)$	–
$\delta k_{xz}^{a}$	$\frac{g\cos\left(2\omega_{r}T\right)}{2}$	–	$-\frac{g\sin\left(2\omega_{r}T\right)}{2}$
$\delta k_{zx}^{a}$	$\frac{g\cos\left(2\omega_{r}T\right)}{2}$	–	$-\frac{g\sin\left(2\omega_{r}T\right)}{2}$
$\delta k_{yy}^{g}$	$g\omega_{r}T$	–	–
$\delta k_{xx}^{a}$	$-\frac{g\sin\left(2\omega_{r}T\right)}{2}$	–	$-\frac{g\cos\left(2\omega_{r}T\right)}{2}$
$\nabla_{x}^{I}$	$\cos\left(\omega_{r}T\right)$	–	$-\sin\left(\omega_{r}T\right)$
$\nabla_{z}^{I}$	$\sin\left(\omega_{r}T\right)$	–	$\cos\left(\omega_{r}T\right)$
$\varepsilon_{x}^{I}$	–	$-\frac{g\sin\left(\omega_{r}T\right)}{\omega_{r}}$	–
$\varepsilon_{z}^{I}$	–	$\frac{g\cos\left(\omega_{r}T\right)-g}{\omega_{r}}$	–
$\delta K_{Ax2}^{}$	$g^{2}\sin\left(2\omega_{r}T\right)\sin\left(\omega_{r}T\right)/2$	–	$-g^{2}\sin^{3}\left(\omega_{r}T\right)$
$\delta K_{Az2}^{}$	$g^{2}\sin\left(2\omega_{r}T\right)\cos\left(\omega_{r}T\right)/2$	–	$g^{2}\cos^{3}\left(\omega_{r}T\right)$
$\delta r_{x}^{I}$	${\left(\omega_{r}\right)}^{2}\cos\left(\omega_{r}T\right)$	–	${\left(\omega_{r}\right)}^{2}\sin\left(\omega_{r}T\right)$
$\delta r_{z}^{I}$	$-{\left(\omega_{r}\right)}^{2}\sin\left(\omega_{r}T\right)$	–	${\left(\omega_{r}\right)}^{2}\cos\left(\omega_{r}T\right)$

**Table 2:** Rotation path of self-calibration process.

Time	Rotation Axis (Inner (I) (*z*-Axis of IMU)/Outer (O) (*x*-Axis of IMU))	Rotation Angle along I/O Axis	Attitude after Rotation (XYZ) (East-North-Upward)
0 s	-	-	ENU
180 s	O	+90${}^{\circ }$	EUS
270 s	O	+180${}^{\circ }$	EDN
360 s	O	+180${}^{\circ }$	EUS
450 s	I	+90${}^{\circ }$	UWS
540 s	I	+180${}^{\circ }$	DES
630 s	I	+180${}^{\circ }$	UWS
720 s	O	+90${}^{\circ }$	SWD
810 s	O	+180${}^{\circ }$	NWU
900 s	O	+180${}^{\circ }$	SWD
990 s	O	+90${}^{\circ }$	DWN
1080 s	O	+90${}^{\circ }$	NWU
1170 s	O	+90${}^{\circ }$	UWS
1260 s	I	+90${}^{\circ }$	WDS
1350 s	I	+90${}^{\circ }$	DES
1440 s	I	+90${}^{\circ }$	EUS
1530 s	O	+90${}^{\circ }$	ESD
1620 s	O	+90${}^{\circ }$	EDN
1710 s	O	+90${}^{\circ }$	ENU

**Table 3:** The observability degree of each state.

State Variable	Singular Value	State Variable	Singular Value
$\delta K_{Ax2}^{}$	232.6811	$\delta K_{Ay2}^{}$	197.5225
$\delta K_{Az2}^{}$	51.2133	$\delta r_{x}^{I}$	43.6781
$\delta r_{x}^{I}$	30.7752	$\delta r_{x}^{I}$	22.1765

**Table 4:** Estimation results of different methods.

Estimated Parameters	Proposed Method	Traditional Method	Reference Values
$\varepsilon_{x}$	−0.01345 ${}^{\circ }$/h	−0.03953 ${}^{\circ }$/h	−0.01955 ${}^{\circ }$/h
$\varepsilon_{y}$	0.012587 ${}^{\circ }$/h	0.04478 ${}^{\circ }$/h	0.01685 ${}^{\circ }$/h
$\varepsilon_{z}$	0.04521 ${}^{\circ }$/h	0.1023 ${}^{\circ }$/h	0.04002 ${}^{\circ }$/h
$\nabla_{x}$	412.23 μg	415.22 μg	412.75 μg
$\nabla_{y}$	−812.36 μg	−813.56 μg	−813.74 μg
$\nabla_{z}$	694.25 μg	691.57 μg	695.12 μg
$\delta k_{xx}^{g}$	206,263.25 ${}^{\circ }$/h/pulse	206,269.44 ${}^{\circ }$/h/pulse	206,263.25 ${}^{\circ }$/h/pulse
$\delta k_{yy}^{g}$	206,269.34 ${}^{\circ }$/h/pulse	206,260.98 ${}^{\circ }$/h/pulse	206,268.87 ${}^{\circ }$/h/pulse
$\delta k_{zz}^{g}$	206,267.22 ${}^{\circ }$/h/pulse	206,268.74 ${}^{\circ }$/h/pulse	206,267.84 ${}^{\circ }$/h/pulse
$\delta k_{xx}^{a}$	98,021.66 m/$s^{2}$/pulse	98,020.97 m/$s^{2}$/pulse	98,021.39 m/$s^{2}$/pulse
$\delta k_{yy}^{a}$	98,015.23 m/$s^{2}$/pulse	98,019.37 m/$s^{2}$/pulse	98,015.54 m/$s^{2}$/pulse
$\delta k_{zz}^{a}$	98,036.94 m/$s^{2}$/pulse	98,031.52 m/$s^{2}$/pulse	98,036.56 m/$s^{2}$/pulse
$\delta k_{yx}^{g}$	3.547′	4.125′	3.368′
$\delta k_{zx}^{g}$	−2.365′	−3.122′	−2.674′
$\delta k_{zy}^{g}$	11.245′	11.544′	10.941′
$\delta k_{xy}^{a}$	9.124′	9.426′	9.221′
$\delta k_{xz}^{a}$	7.586′	7.138′	7.225′
$\delta k_{yx}^{a}$	1.747′	1.529′	1.596′
$\delta k_{yz}^{a}$	−5.618′	−5.221′	−5.625′
$\delta k_{zx}^{a}$	3.027′	3.291′	3.171′
$\delta k_{zy}^{a}$	6.107′	5.822′	6.128′
$\delta K_{Ax2}$	−5.754 ppm	−7.225 ppm	−5.551 ppm
$\delta K_{Ay2}$	23.485 ppm	29.569 ppm	21.993 ppm
$\delta K_{Az2}$	30.241 ppm	27.226 ppm	30.453 ppm

## Data Availability

Not applicable.
